# Macular choroidal thickness in highly myopic women during pregnancy and postpartum: a longitudinal study

**DOI:** 10.1186/s12884-018-1865-3

**Published:** 2018-06-08

**Authors:** Wei Chen, Li Li, Hongyuan Zhang, Yan Li, Xu Chen, Yue Zhang

**Affiliations:** 1 Clinical College of Ophthalmology, Tianjin Medical University, No.4, Gansu Road, Tianjin City, People’s Republic of China; 2 Tianjin Central Hospital of Gynecology and Obstetrics, Tianjin, China

**Keywords:** Macular CT, Highly myopic pregnancy, Enhanced depth imaging optical coherence tomography, Postpartum

## Abstract

**Background:**

High myopia, a cause of serious visual impairment, is a significant global public health concern. We investigate longitudinal changes in macular choroidal thickness (CT) during pregnancy and 6-months postpartum in women with high myopia (HM).

**Methods:**

A prospective longitudinal study was conducted in HM-pregnant women during the course of pregnancy (*n* = 42 eyes, 42 patients) and 6 months postpartum (*n* = 40 eyes, 40 patients, two cases lost).Macular CT was measured via enhanced-depth imaging (EDI)-optical coherence tomography (OCT) (EDI-OCT). Intraocular pressure (IOP), axial length (AL), refractive error, mean arterial pressure (MAP), mean ocular perfusion pressure (MOPP), and body mass index (BMI) were also measured.

**Results:**

Macular CTs of HM pregnant women (214.3 ± 52.3 μm) had increased significantly during the third trimester of pregnancy compared with postpartum women (192.7 ± 51.9 μm, *p* = 0.014). No significant differences in AL, refractive error, or MAP were found between pregnant and postpartum groups (*p* > 0.05 for all parameters).During pregnancy, macular CT was negatively correlated with AL (first trimester: *p* = 0.010; second trimester: *p* = 0.013; and third trimester: *p* = 0.008) and positively correlated with refractive error (first trimester: *p* = 0.038; second trimester: *p* = 0.024; and third trimester: *p* = 0.010). No correlations between macular CT and age, IOP, MOPP, MAP, or BMI were found.

**Conclusions:**

Our study revealed the presence of a significantly thicker choroid during the third trimester of pregnancy compared with 6-mo postpartum in HM women. Macular CT positively correlated with refractive error and negatively correlated with AL during pregnancy, but did not correlate with gestational age, MOPP, IOP, MAP, or BMI.

## Background

As the worldwide prevalence of myopia increases, the proportion of patients with high myopia (HM) is now as high as ~ 4% in the adult Asian population [1]. Thus, the possibility exists that there will be an increase in pregnant women with HM in China. HM, which is defined as a myopic refractive error of > 6 diopters (D) or an axial length (AL) of > 26.5 mm, is accompanied by characteristic pathological changes, including chorioretinal myopic atrophy, i.e., thinning of the choroid and retina [2]. This debilitating condition results in photoreceptor cell death and is accompanied by an irreversible and progressive loss of central visual function. HM pregnant women often discuss ocular changes as their first healthy problem with their obstetricians, therefore, awareness of these fundus changes is important to better care for HM pregnant patients.

Enhanced depth imaging (EDI)-optical coherence tomography (OCT) (EDI-OCT) is a recently developed technique that provides high-quality images of the choroid [3]. Thus, EDI-OCT measurements of choroidal thickness (CT) is useful for observing choroidal changes in HM pregnant women. Using EDI-OCT, Kara et al. demonstrated that subfoveal CT increases in pregnant women compared with age-matched non-pregnant women, but provided no longitudinal data [4]. However, Takahashi et al. demonstrated that CTs did not differ significantly between pregnant and non-pregnant women [5]. While several studies have focused on measuring CT in HM persons or pregnant women [4, 6], none have done so in HM women during pregnancy. Thus, the goal of our study was to know the morphological changes of choroid during the course of pregnancy in HM women longitudinally.

During pregnancy, the maternal cardiovascular system undergoes profound adaptive hemodynamic changes, including increased cardiac output and stroke volume, and reduced blood pressure and peripheral vascular resistance [7]. Vascular blood flow relies on perfusion pressure (PP), and mean ocular perfusion pressure (MOPP) is considered as the driving force of ocular blood flow [8]. Considering the highly vascular nature of the choroid, we hypothesized that MOPP and intraocular pressure (IOP) are major factors associated with CT in HM pregnancy, and that correlations exist between CT and MOPP and between CT and IOP during pregnancy. Thus, we also sought to determine the key factors associated with CT during pregnancy. Furthermore, since studies of refractive error changes are conflicting in their findings, we also chose to investigate such changes during HM pregnancy [9, 10].

We investigated the longitudinal changes in CT in HM women during pregnancy and 6 months postpartum via EDI-OCT, and evaluated the relationships between macular CT and age, AL, refractive error, IOP, mean arterial pressure (MAP), MOPP, and body mass index (BMI).

## Methods

### Subjects

Recruitment and follow-up were conducted between June 2013 and June 2015 at Tianjin Central Hospital of Gynecology Obstetrics and Tianjin Eye Hospital, Tianjin, China. Although patients with bilateral HM were enrolled in the study, only the eye with higher myopia was studied. Tianjin Central Hospital of Gynecology Obstetrics is a major child birth hospital in Tianjin, China, overseeing delivery of > 10,000 babies annually. Written consent from each patient was obtained at recruitment. The study followed the tenets of the Declaration of Helsinki and was approved by the local ethics committee.

A total of 42 HM pregnant women (*n* = 42 eyes) were recruited during their first antenatal visit (11 weeks gestation). Inclusion criteria for the study comprised healthy patients with eyes of AL > 26.5 mm (biometric definition, as previously suggested [11]), refractive error > 6 D, no apparent macular abnormalities (e.g., choroidal neovascularization or macular holes), and aged 25–35 years. Exclusion criteria comprised pregnancy-induced complications (e.g., pre-eclampsia), previous ocular surgery, use of immunosuppressive drugs, glaucoma, retinal detachment, or eyes with poorly visualizable chorioscleral interfaces measured by EDI-OCT.

Patients were scheduled for four examinations each during gestation weeks 11–12 (first trimester group), gestation weeks 22–24 (second trimester group), gestation weeks 32–34 (third trimester group), and postpartum month ~ 6 (postpartum group). As two cases were lost to follow-up at 6-months postpartum, the postpartum group comprised 40 patients (*n* = 40 eyes).

### Examinations

All participants underwent full ophthalmic examinations (including assessment of visual acuity (VA), refractive error, and IOP, AL measurements, and optic nerve head evaluation with a 90-D lens.

IOP was measured by noncontact tonometry (TX-20 model) (Canon, Tokyo, Japan) at the time of OCT imaging, and the average of three measurements was used for analysis. Spherical equivalents (SE) of refractive error were measured by autorefractometry (RK-3) (Canon, Tokyo, Japan) and ALs, by interferometry (IOL-Master) (Carl Zeiss Meditec, Dublin, CA, USA). Again, the average of three measurements was used for analysis.

Standard protocol blood pressure (BP) measurements were taken on the upper right arm using the automatic Omron HEM 705CP sphygmomanometer (Omron Healthcare Inc., Lake Forest, IL, USA) following a 5-min rest period [12]. Three separate measurements were averaged for analysis. Measurements were performed with the patient in a seated, resting position just before EDI-OCT imaging. MOPP was calculated according to the following equation: MOPP = (2/3 × MAP - IOP) [13], where MAP = diastolic BP + 1/3 × (systolic BP- diastolic BP) [14].

Body weight and height (bare feet) were measured to an accuracy of 0.1 kg and 0.1 cm, respectively, using an adjusted weighing machine and a height measuring instrument. Both measurements were taken separately and measured twice. If the first two measurements differed by 1.0 cm (height) or 200 g (weight), a third measurement was taken and included in the average calculation. BMI was calculated as weight divided by height squared (kg/m^2^).

The scan protocol of the Cirrus OCT (Carl Zeiss Meditec, Jena, Germany) generates a cube of data through a 9-mm line around the macula via its HD 5-line raster mode [15]. Using the caliper system provided by the software, CT was measured from the outer surface of the hyper-reflective retinal pigment epithelium (RPE) line to the hyper-reflective line of the inner scleral border. CTs at the fovea, and 3 mm superiorly, inferiorly, temporally, and nasally to the fovea were measured between 9:00 a.m. and 12:00 p.m. on test days. The mean overall CT was obtained by calculating average values of CTs at all locations and was recorded as macular CT. Two independent observers manually measured each CT. Measurements obtained from two observers were averaged for analysis.

### Statistical analyses

Statistical analyses were performed using version 17.0 SPSS software (SPSS, Inc., Chicago, IL, USA). All data are reported as mean ± standard deviation (SD), with a 95% confidence interval (CI). An unpaired *t*-test was used to compare variables between various groups (with normal distribution); the Mann–Whitney U-test was used to compare variables between various groups (without normal distribution). To examine the association between macular CT variables and age, AL, refractive error, IOP, MOPP, MAP, and BMI, the multiple regression analysis were calculated. A *p* < 0.05 was considered statistically significant.

## Results

### Demographics and clinical features

Significant differences were seen in age (all *p* < 0.05, postpartum group compared with the first, and second trimesters groups), IOP and BMI (both *p* < 0.01, postpartum group compared with the third trimester group), MOPP and macular CT (both *p* < 0.05, postpartum group compared with the third trimester), while no significant differences in AL, refractive error, or MAP were found between pregnant, and postpartum groups (all values of *p* > 0.05) (Table 1).

**Table 1:** Demographics and Clinical Characteristics of the patients throughout pregnancy and 6-months postpartum

Variables	Groups			
	First trimester (*n* = 42)	Second trimester (*n* = 42)	Third trimester (*n* = 42)	Postpartum (*n* = 40)
Mean Age (years)	28.5 ± 1.7	28.9 ± 1.7	29.3 ± 1.6	29.7 ± 1.7^a^
AL (mm)	27.6 ± 0.7	27.6 ± 0.7	27.6 ± 0.7	27.6 ± 0.7
Refractive error (D)	−9.7 ± 1.4	− 9.7 ± 1.4	−9.8 ± 1.4	− 9.6 ± 1.4
IOP (mmHg)	15.4 ± 2.8	15.0 ± 2.4	14.4 ± 2.8	15.8 ± 2.8^b^
MOPP (mmHg)	38.8 ± 3.3	39.0 ± 3.2	39.7 ± 2.8	38.6 ± 3.1^c^
MAP (mmHg)	81.7 ± 8.1	82.1 ± 7.9	82.7 ± 7.8	81.6 ± 7.9
BMI (kg/m^2^)	21.9 ± 2.0	22.7 ± 2.0	24.4 ± 1.9	22.3 ± 2.2^b^
Macular CT (μm)	194.5 ± 50.3	201.9 ± 51.5	214.3 ± 52.3	192.7 ± 51.9^c^

*AL* axial length, *D* diopters, *IOP* intraocular pressure, *MOPP* mean ocular perfusion pressure, *MAP* mean arterial pressure, *BMI* body mass index, *CT* choroidal thickness

^a^*p <* 0.05 compared with the first, and second trimesters by *t*-test; ^b^*p <* 0.01 compared with the third trimester by *t*-test; ^c^*p <* 0.05 compared with the third trimester by *t*-test

**Fig. 1:**
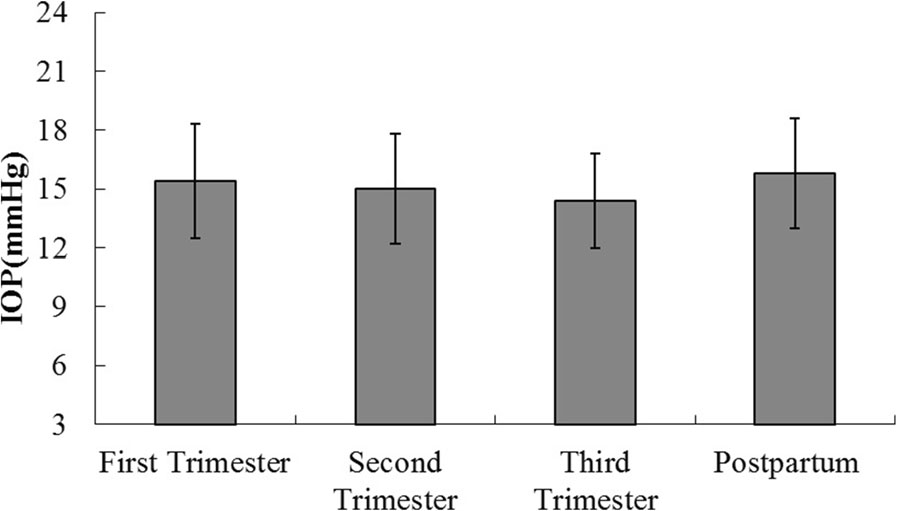
IOP levels in study groups. Results are geometric mean 95% CI. The third trimester group had lower IOP compared to the first trimester group (*p* = 0.02) and the postpartum group (*p* = 0.004)

IOPs were significantly lower in the third trimester group compared to first trimester (*p* = 0.02), and postpartum (*p* = 0.004) groups, but not when compared to the second trimester (*p* > 0.05) group. There were no significant differences in IOPs observed between any other two groups (all *p* > 0.05) (Fig. 1).

**Fig. 2:**
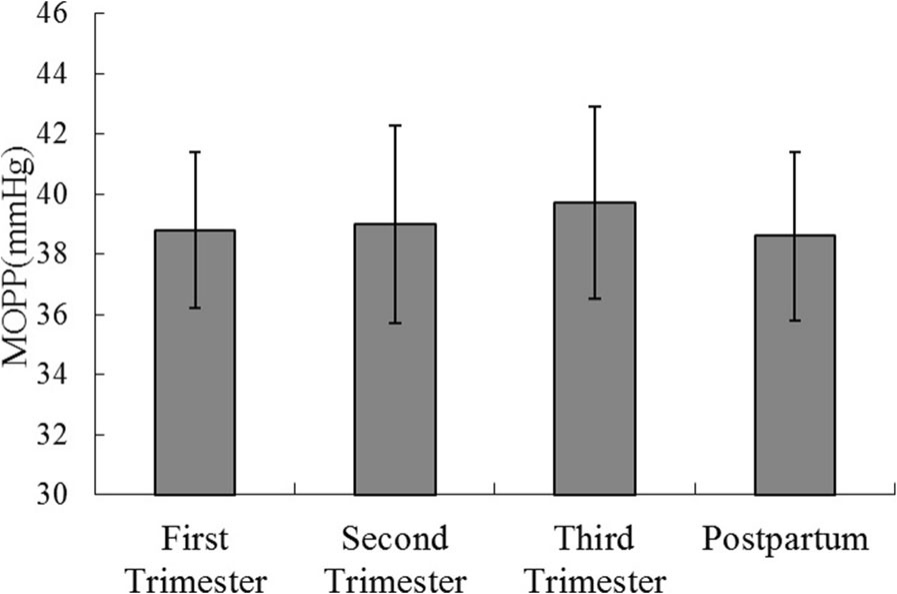
MOPP levels in study groups. Results are geometric mean 95% CI; The third trimester group had higher MOPP compared to the postpartum group (*p* = 0.035)

MOPPs increased throughout pregnancy, and was significantly higher in the third trimester group than in the postpartum (*p* = 0.035) groups, while no differences were found in the first (*p* > 0.05) and second trimester (*p* > 0.05) groups. There were no significant difference in MOPPs between any other two groups (all *p* > 0.05) (Fig. 2).

BMIs were significantly higher in the third trimester group than in the first, second, postpartumgroups (all *p* < 0.001), while no significant differences were found between any other two groups (all *p* > 0.05).

### Macular CT

**Fig. 3:**
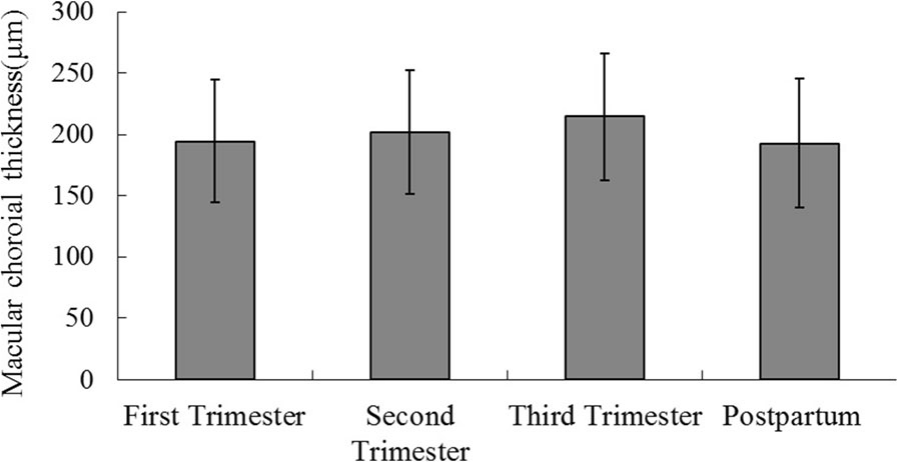
Macular CT levels in study groups. Results are geometric mean 95% CI; The third trimester group had thicker CT compared to the postpartum group (*p* = 0.014)

Macular CTs increased significantly in the third trimester group compared to the postpartum (*p* = 0.014) groups, while no significant differences were observed in the first and second trimester groups (both *p* > 0.05); There were no significant differences in macular CT between any other two groups (all *p* > 0.05) (Fig. 3).

### Correlation of macular CT with other variables

In studying the correlation between macular CT and measured variables, we found a significantly negative correlation with AL: (*p* = 0.01 in the first trimester; *p* = 0.013 in the second trimester; and *p* = 0.008 in the third trimester), while there was a strong positive correlation with refractive error: (*p* = 0.038 in the first trimester; *p* = 0.024 in the second trimester; and *p* = 0.01 in the third trimester). However, no significant correlations were found between macular CT and age, IOP, MOPP, MAP, or BMI during pregnancy (all *p* > 0.05) (Table 2).

**Table 2:** Correlations between macular CT and clinical and demographic factors

Factors	First trimester		Second trimester		Third trimester	
	Coefficient	*p* value	Coefficient	*p* value	Coefficient	*p* value
Age	−0.116	0.982	−1.297	0.791	−0.827	0.866
AL	−8.621	0.01	−8.268	0.013	−8.932	0.008
Refractive error	12.484	0.038	13.822	0.024	16.335	0.010
IOP	−2.398	0.597	−4.821	0.332	−4.181	0.422
MOPP	−7.430	0.060	−7.225	0.063	−7.676	0.058
MAP	−0.561	0.814	−1.579	0.712	−1.237	0.769
BMI	−6.614	0.205	−5.442	0.314	−5.623	0.315

*CT* choroidal thickness, *AL* axial length, *IOP* intraocular pressure, *MOPP* mean ocular perfusion pressure, *MAP* mean arterial pressure, *BMI* body mass index

## Discussion

CT is a significant indicator of fundus lesions in HM [16]. In the present study, we found that macular CTs in the third trimester of pregnancy reached their maximum values (214.3 ± 52.3 μm), which were significantly greater compared to the postpartum group (192.7 ± 51.9 μm; *p* = 0.014). However, there were no differences compared with the first and second trimester (*p* > 0.05) groups, indicating that CTs didn’t change significantly throughout pregnancy (Fig. 3). Such a finding is consistent with those of Kara et al. for normal pregnant women [4]. However, Takahashi et al. found that CTs did not differ significantly between pregnant and non-pregnant women [5]. The increases in CT may be due to an increased release of vasodilatory signaling molecules, e.g., nitric oxide, prostacyclin, and prostaglandins, all of which are key elements in the physiological systemic vasodilatation of pregnancy [17]. Our study provides the first in vivo demonstration of increased CT in HM pregnancy, including longitudinal data.

We found no significant differences in refractive errors in any of the trimester or postpartum groups (*p* > 0.05). Refractive changes during pregnancy are somewhat controversial. Mehdizadehkashi K et al. followed 107pregnant women throughout the course of their pregnancies and 3 months postpartum, and reported no significant changes in refractive errors [9], findings identical to ours. Imafidon reported a ~ 1.25D myopic shift, attributing it to lens hydration [10]. Pizzarello reported that 15% of the women who complained of visual changes had experienced a myopic shift from pre-pregnancy levels [18]. We found a significant correlation between macular CT and refractive error during pregnancy (*p* < 0.05), indicating that choroidal thinning is prominent in highly myopic eyes, a finding consistent with another study of our group in non-pregnant highly myopic patients [15].

IOP can become lower throughout pregnancy [19]. We not only found IOP to be decreased during pregnancy, but also significantly so in the third trimester compared with first trimester, and postpartum groups (Fig. 1). The reasons for this are attributed to reduced episcleral venous pressure, greater aqueous outflow, and the effects of progesterone [20]. Similar investigations of pregnant women have shown a significant decrease in IOP compared with non-pregnant women [21]. Akar et al. also found that decreases in IOPs were greater during the third trimester [22]. In our study, we found no correlation between macular CT and IOP during pregnancy (*p* > 0.05).

Although the primary regulatory role of the choroid is well known, the in vivo clinical association of CT with MOPP has yet to be determined in HM pregnancy. Previous studies have reported that ocular blood flow increases throughout gestation [23]. In our study, we found that MOPP increased during the third trimester compared with postpartum groups (*p* < 0.05) (Fig. 2). However, no significant associations between CT and MOPP during pregnancy (*p* > 0.05) (Table 2) were found. These findings are in accordance with those of Kim et al., who found no significant correlation between subfoveal CT and MOPP (*p* > 0.05) in healthy subjects [24]. In contrast, Sayin et al. found subfoveal CT to be positively correlated with MOPP in pregnant women with no preeclampsia, while no correlation existed in pregnant women with pre-eclampsia [25]. The reasons for there being no significant correlation between macular CT and MOPP in our study may be the following. First, autoregulation of choroidal blood flow causes only slight changes in CT within normal ranges of IOP and ocular perfusion pressure (OPP) in healthy pregnant subjects. Second, the increase in CT may be secondary to the increase and accumulation of intrachoroidal or intrachoroidal cellular fluid substances.

Macular CT showed a negative correlation with AL (*p* < 0.05) throughout pregnancy (Table 2). These results are similar to those of Nishida et al. in their study of HM myopic eyes [26]. Meanwhile we found no significant association between macular CT and pregnancy-related factors, e.g. gestational age, BMI, or MAP (all *p* > 0.05) (Table 2).

Potential limitations to our study are as follows. First, the overall number of enrolled patients was relatively small. Large-scale studies are recommended in the future. Second, we were not able to measure all parameters before pregnancy in all patients to obtain longitudinal data for the whole study population. Third, there was some selection bias, as pregnant women were recruited from only one site. However, such a bias is not expected to be differential in nature. Fourth, CT measurements were performed manually, as there exist no precise automated software which only can reduce time and labor cost for measuring CT [27]. In the present study, two masked observers performed measurements, with adjudication if there was a difference of > 5% in agreement of measurements.

## Conclusions

Our study demonstrates the presence of a significantly thicker choroid during the third trimester of pregnancy of HM women compared to 6-months after delivery. Macular CT positively correlated with refractive error and negatively correlated with AL during pregnancy, but did not correlate with gestational age, MOPP, IOP, MAP, or BMI. Improved in vivo visualization of the choroid and measurement of CT using EDI-OCT will go far to improve our understanding of the morphologic changes occurring in the choroid in HM pregnancies.

## Abbreviations

AL: Axial length
BMI: Body mass index
BP: Blood pressure
CI: Confidence interval
CT: Choroidal thickness
EDI-OCT: Enhanced-depth imaging (EDI)-optical coherence tomography
HM: High myopia
IOP: Intraocular pressure
MAP: Mean arterial pressure
MOPP: Mean ocular perfusion pressure
OPP: Ocular perfusion pressure
PP: Perfusion pressure
RPE: Retinal pigment epithelium
SD: Standard deviation
SE: Spherical equivalents
VA: Visual acuity

## References

[1] Kim EC, Morgan IG, Kakizaki H, Kang S, Jee D (2013). Prevalence and risk factors for refractive errors: Korean National Health and nutrition examination survey 2008-2011. PLoS One.

[2] You QS, Peng XY, Xu L, Chen CX, Wang YX, Jonas JB (2014). Myopic maculopathy imaged by optical coherence tomography: the bei jing eye study. Ophthalmology.

[3] Dhoot DS, Huo S, Yuan A (2013). Evaluation of choroidal thickness in retinitis pigmentosa using enhanced depth imaging optical coherence tomography. Br J Ophthalmol.

[4] Kara N, Sayin N, Pirhan D (2014). Evaluation of subfoveal choroidal thickness in pregnant women using enhanced depth imaging optical coherence tomography. Curr Eye Res.

[5] Takahashi J, Kado M, Mizumoto K, Igarashi S, Kojo T (2013). Choroidal thickness in pregnant women measured by enhanced depth imaging optical coherence tomography. Jpn J Ophthalmol.

[6] Fujiwara T, Imamura Y, Margolis R, Slakter JS, Spaide RF (2009). Enhanced depth imaging optical coherence tomography of the choroid in highly myopic eyes. Am J Ophthalmol.

[7] Melchiorre K, Sharma R, Thilaganathan B (2012). Cardiac structure and function in normal pregnancy. Curr Opin Obstet Gynecol.

[8] Schmidl D, Garhofer G, Schmetterer L. The complex interaction between ocular perfusion pressure and ocular blood flow-relevance for glaucoma. Exp Eye Res. 2011;93:141–55. 10.1016/j.exer.2010.09.002 20868686

[9] Mehdizadehkashi K, Chaichian S, Mehdizadehkashi A (2014). Visual acuity changes during pregnancy and postpartum: a cross-sectional study in Iran. J Pregnancy.

[10] Imafidon CO, Imafidon JE (1992). Contact lenses in pregnancy. Br J Obstet Gynecol.

[11] Chen W, Guan Y, He G (2015). Aqueous Levels of Pigment Epithelium-Derived Factor and Macular Choroidal Thickness in High Myopia. J Ophthalmol.

[12] Vera-Cala LM, Orostegui M, Valencia-Angel LI, López N, Bautista LE (2011). Accuracy of the Omron HEM-705 CP for blood pressure measurement in large epidemiologic studies. Arq Bras Cardiol.

[13] Rishi P, Rishi E, Mathur G, Raval V (2013). Ocular perfusion pressure and choroidal thickness in eyes with polypoidal choroidal vasculopathy, wet-age-related macular degeneration, and normals. Eye (Lond).

[14] Skråstad RB, Hov GG, Blaas HG, Romundstad PR, Salvesen KÅ (2014). A prospective study of screening for hypertensive disorders of pregnancy at 11 to 13 weeks in a Scandinavian population. Acta Obstet Gynecol Scand.

[15] Chen W, Song H, Xie S, Han Q, Tang X, Chu Y (2015). Correlation of macular choroidal thickness with concentrations of aqueous vascular endothelial growth factor in high myopia. Curr Eye Res.

[16] Wang NK, Lai CC, Chu HY (2012). Classification of early dry-type myopic maculo- pathy with macular choroidal thickness. Am J Ophthalmol.

[17] Clapp JF, Capeless E (1997). Cardiovascular function before, during, and after the first and subsequent pregnancies. Am J Cardiol.

[18] Pizzarello LD (2003). Refractive changes in pregnancy. Graefes Arch Clin Exp Ophthalmol.

[19] Efe YK, Ugurbas SC, Alpay A, Ugurbas SH (2012). The course of corneal and intraocular pressure changes during pregnancy. Can J Ophthalmol.

[20] Grant AD, Chung SM (2013). The eye in pregnancy: ophthalmologic and neuro-ophthalmologic changes. Clin Obstet Gynecol.

[21] Ebeigbe JA, Ebeigbe PN, Ighoroje AD (2011). Intraocular pressure in pregnant and non-pregnant Nigerian women. Afr J Reprod Health.

[22] Akar Y, Yucel I, Akar ME, Zorlu G, Ari ES (2005). Effect of pregnancy on intraobserver and intertechnique agreement in intraocular pressure measurements. Ophthalmologica.

[23] Centofanti M, Migliardi R, Bonini S, Manni G, Bucci MG, Pesavento CB (2002). Pulsatile ocular blood flow during pregnancy. Eur J Ophthalmol.

[24] Kim M, Kim SS, Kwon HJ, Koh HJ, Lee SC (2012). Association between choroidal thickness and ocular perfusion pressure in young, healthy subjects: enhanced depth imaging optical coherence tomography study. Invest Ophthalmol Vis Sci.

[25] Sayin N, Kara N, Pirhan D, Vural A, Araz Ersan HB, Tekirdag AI (2014). Subfoveal choroidal thickness in preeclampsia: comparison with normal pregnant and nonpregnant women. Semin Ophthalmol.

[26] Nishida Y, Fujiwara T, Imamura Y, Lima LH, Kurosaka D, Spaide RF (2012). Choroidal thickness and visual acuity in highly myopic eyes. Retina.

[27] Lee S, Fallah N, Forooghian F, Ko A, Pakzad-Vaezi K, Merkur AB (2013). Comparative analysis of repeatability of manual and automated choroidal thickness measurements in nonneovascular age-related macular degeneration. Invest Ophthalmol Vis Sci.

